# Supplementary material for: Tailoring near-field thermal radiation between metallo-dielectric multilayers using coupled surface plasmon polaritons
Source: Nat Commun. 2018 Oct 16;9:4302. doi: 10.1038/s41467-018-06795-w (PMC6191454; doi:10.1038/s41467-018-06795-w)
Supplement: Supplementary file 1 — Supplementary Information [file 41467_2018_6795_MOESM1_ESM.pdf]

Supplementary Information

Tailoring near-field thermal radiation between metallo-dielectric multilayers using coupled surface plasmon polaritons

Lim *et al.*

Supplementary Note 1. Fabrication process of emitter and receiver parts

Supplementary Figure 1 provides a cross-section transmission electron microscope (TEM) view of the fabricated receiver part. The metallo-dielectric (MD) multilayer (multilayer of Ti/MgF_2) is deposited on the thick Ti film (180 nm). In this MD multilayer, the thicknesses of the Ti and MgF_2 layers are estimated to be 10 nm and 90 nm, respectively (i.e., the volume filling ratio, *f*, is 0.1). Detailed MEMS-fabrication process of emitter and receiver parts is depicted in Supplementary Fig. 2. Quartz (fused silica) substrate with a thickness of 525 μm (emitter) or 1 mm (receiver) is cleaned with a mixture of sulfuric acid (H_2SO_4) and hydrogen peroxide (H_2O_2) at a ratio of 2:1. A doped-Si film (500-nm thick) is deposited on the cleaned quartz substrate by low pressure chemical vapor deposition (LPCVD) (Supplementary Fig. 2a or a′) and a photoresist (PR, AZ9260) is patterned on the doped-Si film using UV lithography (Supplementary Fig. 2b or b′). Then, by means of reactive ion etching (RIE), deposited doped-Si layer is patterned (Supplementary Fig. 2c or c′) and used as etching mask during isotropic wet etching of the quartz substrate with buffered oxide etchant (6:1 BOE) (Supplementary Fig. 2d or d′). The quartz substrates for the emitter and receiver parts are wet etched for three hours and for eight hours to make 12-μm- and 28-μm-deep trenches, respectively. Then, the patterned PR which protects the doped-Si film from being etched during quartz wet etching with BOE is removed with acetone. Right after the acetone step, the emitter or receiver part is rinsed with methanol and running deionized water (Supplementary Fig. 2e or e′). The remaining doped Si is removed with KOH at 95°C (Supplementary Fig. 2f or f′). The aforementioned steps are used to make trenches on the quartz substrate. On these clean and grooved quartz substrates, successive electron-beam (E-beam) evaporation processes are conducted with shadow masks. First, a 190-nm-thick Ti film (serving as a calibration heater) is deposited with a shadow mask which has 700 μm width for the emitter part or 500 μm width for the receiver part (Supplementary Fig. 2g or g′). Then, a 2.19-μm-thick SiO_2 layer is deposited (Supplementary Fig. 2h or h′) with a shadow mask that has a wider width than the shadow mask used for the Ti calibration heater. On the SiO_2 insulation layer, three Ti/MgF_2 unit cells on a thick Ti layer (180 nm) are deposited with a shadow mask (Supplementary Fig. 2i or i′) and this multilayer is defined as the MD multilayer. In the subsequent Au layer deposition steps, a Ti layer functions as an adhesion layer. For the emitter part, a Ti/Au layer (100 nm/100 nm) is deposited to suppress the background radiation (Supplementary Fig. 2j). Also, on the back of the substrate, a Ti/Au (100 nm/100 nm) heater is deposited with a shadow mask (Supplementary Fig. 2k); for the receiver part, a Ti/Au (100 nm/100 nm) temperature sensor is deposited in the same manner (Supplementary Fig. 2k′). After dicing the emitter and receiver parts, the two parts are aligned as shown in Supplementary Fig. 2l.

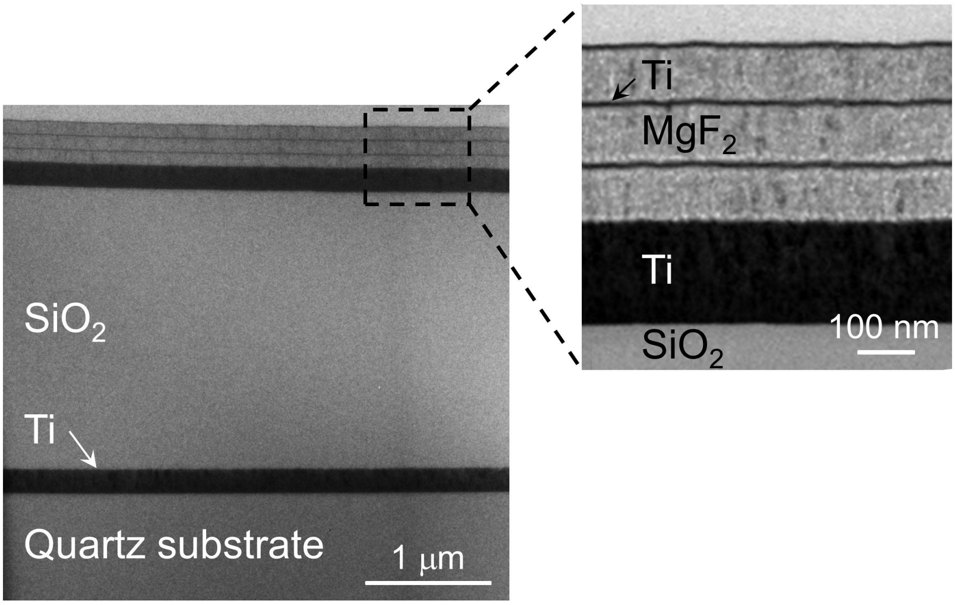

Supplementary Figure 1: Transmission electron microscope (TEM) image of cross-section of fabricated receiver part of microdevice.

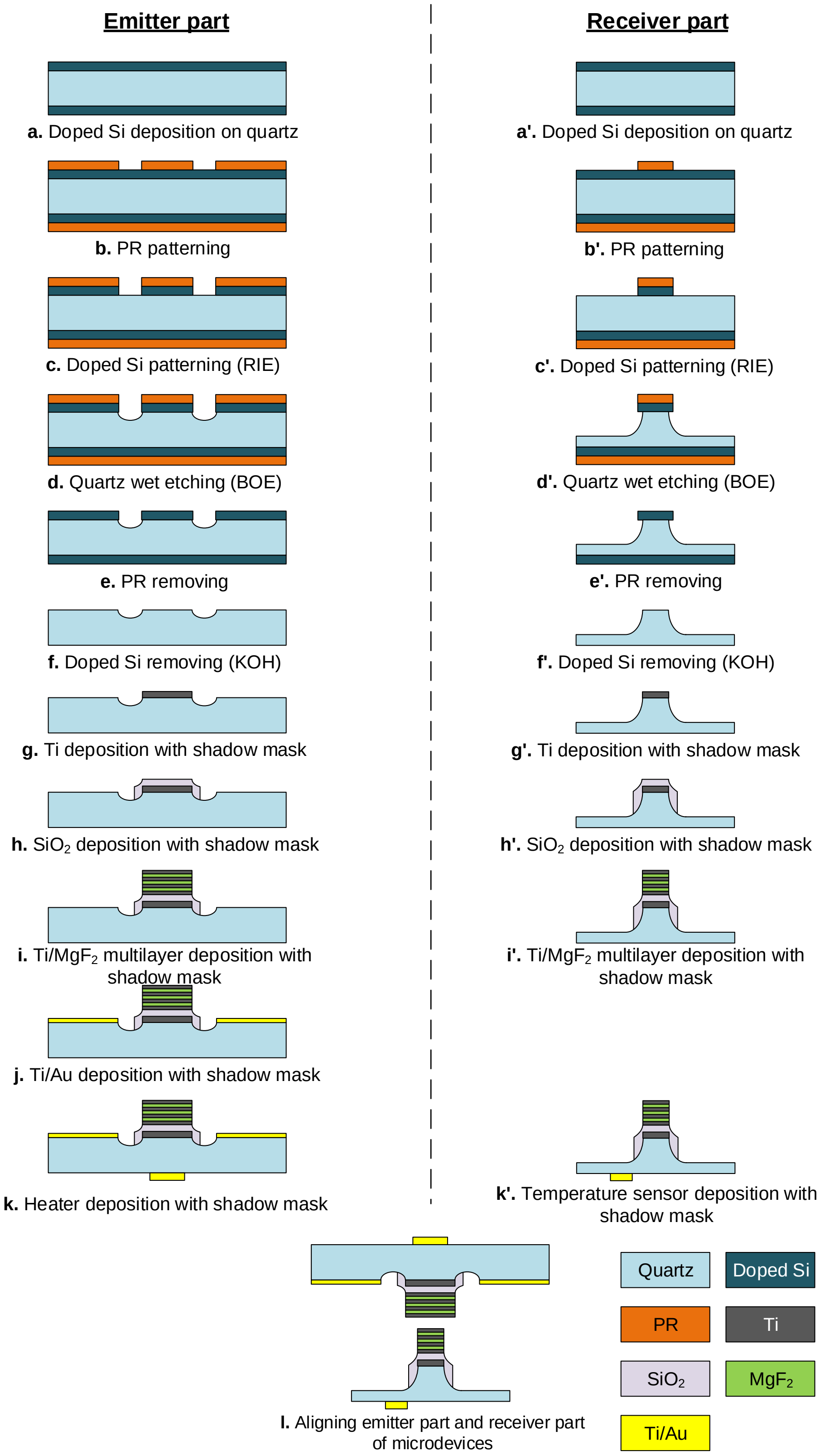

Supplementary Figure 2: MEMS-fabrication process of microdevices. (**a**-**k**) Emitter part fabrication process. (**a′**-**k′**) Receiver part fabrication process. (**l**) Aligned emitter and receiver parts.

Supplementary Note 2. Surface cleaning and characterization

As described in Supplementary Note 1, prior to MEMS-fabrication process, a quartz substrate is cleaned with a mixture of sulfuric acid (H_2SO_4) and hydrogen peroxide (H_2O_2). After removing doped Si (Supplementary Fig. 2f,f′), the surface of the grooved quartz substrate is clean because KOH removes doped Si as well as residual organic materials. During E-beam evaporation processes, the surface can be kept clean because the patterned layers are deposited with shadow masks (Supplementary Fig. 2g-k,g′-k′), without a PR patterning process. Before dicing the fabricated microdevices, the whole surface is coated by the PR to prevent additional contamination. Because this protective PR is just soft baked, it can be completely removed with an appropriate PR stripper (ST-1023). We pour the PR stripper into two separated glass petri dishes and heat them to 60°C on a hot plate. First, the sample is placed in the PR stripper of the first petri dish for 3 minutes. In this step, bulk PR is removed. Then, the sample is put in the second petri dish containing PR stripper for another 3 minutes to remove sidewall PR buildup. Finally, the sample is washed with running deionized water and dried with pure N_2 gas.

After cleaning, the MD-multilayer surface is scanned via a confocal optical profiler (μ-surf, NanoFocus), a stylus line profiler (Dektak-8, Veeco), and an atomic force microscope (AFM, NTEGRA Aura, NT-MDT) to confirm that the cleanliness, planarity, and roughness of the surface are appropriate to perform near-field radiative heat transfer measurement. All experiments were carried out using clean emitter and receiver parts, which have no protrusions higher than 100 nm on the MD-multilayer surfaces. In the insets of Supplementary Fig. 3a,b, scanning results of the MD-multilayer surfaces are plotted. The images are obtained with a confocal optical profiler, which characterizes the cleanliness and planarity of each surface and which has a 800-μm-by-800-μm scan area with 20-nm vertical resolution. The line profile over the width of the active region (i.e., the overlapping area of the MD emitter and receiver), which is illustrated in both confocal optical profiler images (see Supplementary Fig. 3a,b), indicates that the largest difference of deviation from planarity over the width of the active region is about 60 nm for both the emitter and receiver surfaces. The experimental values presented in Fig. 4 of the manuscript are results considering the effect of the planarity on near-field radiative heat transfer between the MD emitter and receiver. In the inset of Supplementary Fig. 3b (confocal optical profile image), because of the optical noise, it seems that there is spike at the edge of the MD receiver surface. Using a stylus line profiler, we confirm that this spike edge does not actually exist. In addition to the line profile over the width of the active region, a line profile along the length of the device is obtained with the stylus line profiler (see Supplementary Fig. 4a,b). As illustrated in Supplementary Fig. 4a, the MD-emitter surface is curved as a concave shape: the largest difference of deviation from planarity is about 210 nm; that of the MD

receiver is estimated at about 110 nm (see Supplementary Fig. 4b). Because the four local gaps are measured from the capacitance and the average vacuum gap distance is obtained using the Derjaguin approximation (see Supplementary note 6), the effect of the curved MD-multilayer surfaces on the near-field radiative heat flux is fully considered. In order to check the roughness, the MD-multilayer surface, with dimensions of 2.5 μm × 2.5 μm is scanned (see Supplementary Fig. 5) via AFM and the height data is stored as a 250 × 250 array. The root-mean-square roughness of the MD-multilayer surface is found to be 3.5 nm, which is extremely small compared to the minimum vacuum gap of 160 nm.

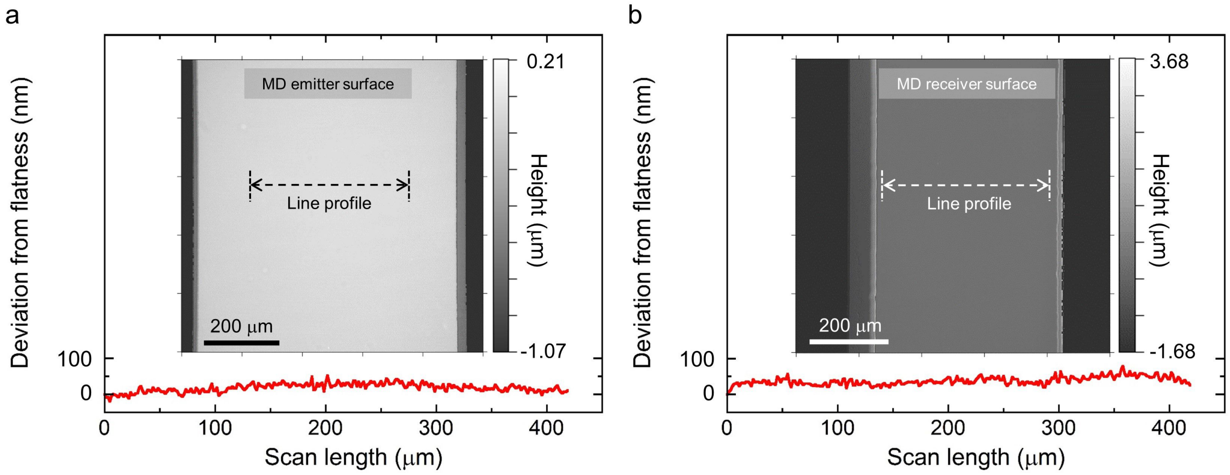

Supplementary Figure 3: Profiles of MD-multilayer surfaces obtained with confocal optical surface profiler over a 800 μm × 800 μm scan area. Inset: confocal optical profile images. (**a**) Emitter part. (**b**) Receiver part.

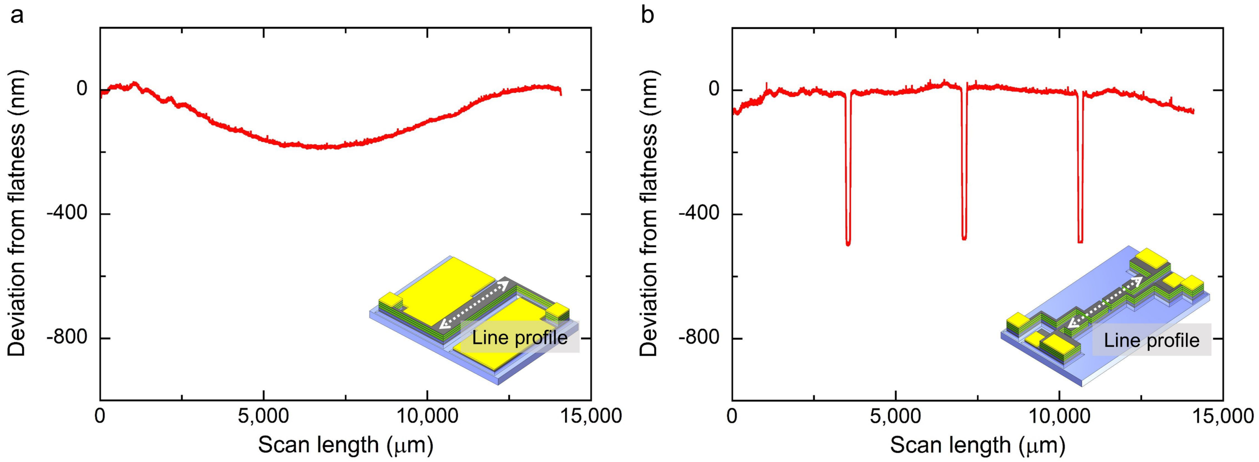

Supplementary Figure 4: Profiles of MD-multilayer surfaces along length of device obtained with stylus line profiler. (**a**) Emitter part. (**b**) Receiver part.

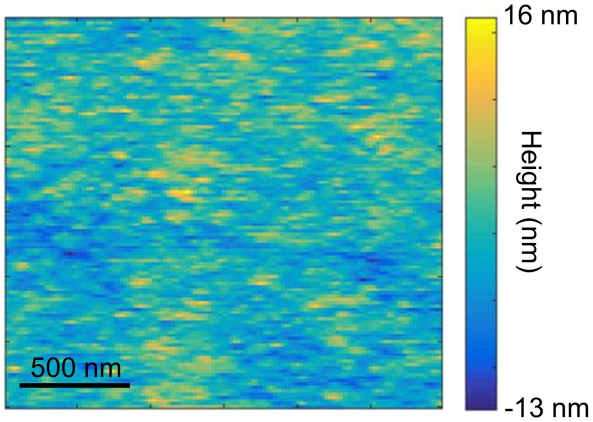

Supplementary Figure 5: AFM image of MD-multilayer surface with scan area of 2.5 μm × 2.5 μm.

Supplementary Note 3. Integrated platform consisting of MEMS-based microdevices and three-axis nanopositioner

In order to control the vacuum gap distance between the MD emitter and the MD receiver at nanoscale, we use a custom-built three-axis nanopositioner composed of three picomotor actuators (8302-V, Newport) and three linear motion guides. Supplementary Figure 6a provides a schematic of the integrated platform, excluding the heat sink and the receiver part, showing the positions of the three picomotor actuators. The photographs of the integrated platform before and after mounting the moving stage of the nanopositioner are shown in Supplementary Fig. 6b,c, respectively. The linear motion guides are employed not only to deliver the motion of the picomotor actuator to the moving stage of the nanopositioner but also to reduce the original moving step of the picomotor actuator by half (< 15 nm).

The emitter and receiver parts are aligned with the three-axis nanopositioner after soldering the electric wires, which are used for temperature and capacitance measurements. The receiver part is fixed on the heat sink located at the center of the three-axis nanopositioner. The emitter part is fixed on the bottom surface of the moving stage of the nanopositioner. In order to achieve precise and consistent alignment of the moving stage of the nanopositioner on the three linear motion guides, a V-groove, a zirconia ball, and a cone-groove are used. On each linear motion guide, an axisymmetric cone-groove is formed and the zirconia ball (diameter: 4 mm) is placed on the cone-groove. On the bottom surface of the moving stage, three V-grooves are formed at the corresponding locations of the zirconia balls. The moving stage of the nanopositioner is then mounted on the three linear motion guides via the three sets of the V-groove, the zirconia ball, and the cone-groove. The alignment of the MD emitter and the MD receiver can be confirmed through the open window of the moving stage. When the MD emitter and the MD receiver are out of alignment, this problem can be corrected by changing the position of the heat sink, which can move independently of the three-axis nanopositioner. After completing the alignment, each displacement made by the three picomotor actuators can change the height and angle of the emitter part leading to variation in the vacuum gap distance between the MD emitter and the MD receiver.

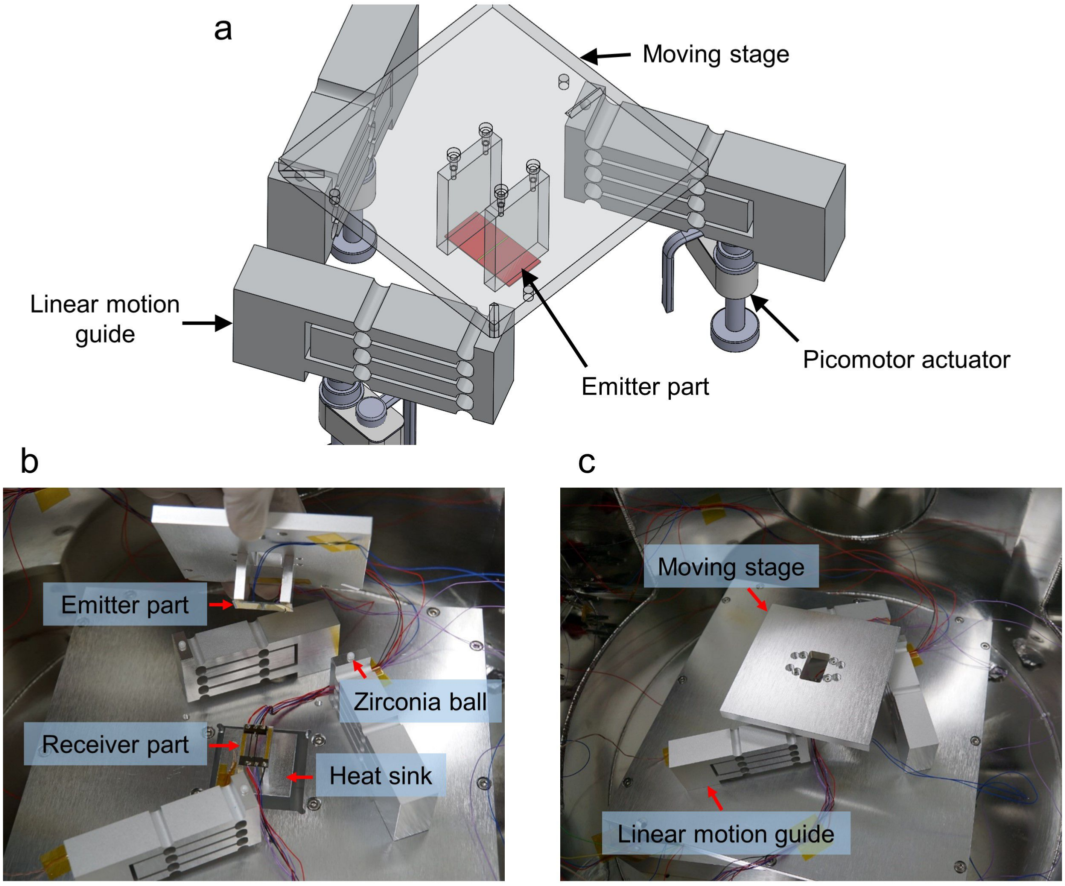

Supplementary Figure 6: Experimental apparatus. (**a**) Schematic of integrated platform. (**b**) Photograph of integrated platform before mounting moving stage of nanopositioner. (**c**) As in **b**, but after mounting.

Supplementary Note 4. Structure of microdevices and data acquisition setup

Supplementary Figure 7a,b are three-dimensional schematics of the MEMS-based microdevices. Each figure shows the patterned metallo-dielectric (MD) multilayer (i.e., Ti/MgF_2 multilayer), the location of each capacitor electrode, and how these electrodes are electrically connected. Please note that the location of the capacitor electrodes, electrical paths, and soldering pads are carefully designed to entirely avoid physical contact between the surfaces of the emitter and receiver parts during measurement.

Supplementary Figure 7a illustrates the configuration of the emitter part of the microdevice. For the MD emitter, the orange-colored area of the topmost Ti layer is used as a capacitor electrode, and the remaining part of the topmost Ti layer functions as an electrical path connecting the capacitor electrode to the soldering pad. On the soldering pads, an Au film is deposited and an electrical wire is soldered to one of the two emitter capacitor soldering pads to connect the MD-emitter-capacitor electrode to an LCR meter (E4980AL, Keysight). To suppress far-field thermal radiation, an Au film is deposited on the remaining area, except for the MD emitter, and an Au heater is located on the backside of the emitter part (see Supplementary Fig. 8).

Supplementary Figure 7b shows the configuration of the receiver part of the microdevice. Beneath the MD receiver, a Ti layer functioning as a calibration heater is deposited on a grooved quartz wafer. Unlike the emitter part, the MD receiver is divided into four segments (illustrated as four orange-colored capacitor electrodes in Supplementary Fig. 7b) to measure the vacuum gap distance as well as the curvature (and parallelism). The four segments of the MD multilayer are electrically isolated by an SiO_2 insulation layer deposited between the Ti calibration heater and the MD multilayer. Patterned Au films are deposited on the four capacitor-soldering pads and calibration-heater-soldering pads. Each capacitor electrode in the receiver part has its own capacitor-soldering pad to connect it to the LCR meter independently through soldered electrical wires. The Ti calibration heater has separate soldering pads that are electrically isolated from the capacitor-soldering pads, such that Joule heating power can be safely applied to the Ti calibration heater during heat-flux calibration (see Supplementary Note 9). The temperature sensor is deposited on the backside of the receiver part (see Supplementary Fig. 8).

When the emitter part and the receiver part are aligned in the three-axis nanopositioner (see Supplementary Note 3), the topmost Ti layers of the MD emitter and the MD receiver overlap (i.e., the orange-colored areas in Supplementary Fig. 7a,b, noted as capacitor electrodes) and thus function as a vacuum gap distance sensor between the MD emitter and the MD receiver. Because the width of the MD-emitter-capacitor electrode (i.e., 720 μm) is larger than that of the MD-receiver-capacitor electrode (i.e., 540 μm), the area of the MD-receiver-capacitor electrodes could be considered as the effective area when converting the measured capacitance to the

vacuum distance (noted as d_1, d_2, d_3, and d_4).

After the calibration of the Au heater and the Au temperature sensor (i.e., resistance temperature detector, Supplementary Note 8), the calibration of heat flux (Supplementary Note 9), and the calibration of capacitance (Supplementary Note 5), vacuum gap distance and corresponding near-field thermal radiative heat flux are measured while moving the MD emitter, which is maintained at constant temperature, close to the MD receiver. Supplementary Figure 8 shows the experimental setup for the measurements of the temperature of the heater (noted as T_{heater}), the vacuum gap distances for the four segments (d_1, d_2, d_3, and d_4), the temperature of the sensor (noted as T_{sen}), and the temperature of the heat sink (noted as T_{TC}).
Using a calibrated result (see Supplementary Note 8), T_{heater} can be estimated from the measured electrical resistance and maintained at the desired value by feedback control of the applied voltage with a sourcemeter (2400 Sourcemeter, Keithley). Using the LCR meter, the four local vacuum gaps (i.e., d_1, d_2, d_3, and d_4) are estimated from the capacitances between the MD-emitter-capacitor electrode and the MD-receiver-capacitor electrodes. When using the three axis nanopositioner to change the position of the emitter part, the local vacuum gaps are controlled to parallelize the MD emitter and the MD receiver (i.e., $d_1 \approx d_4$ and $d_2 \approx d_3$). To prevent electrical interference among the capacitance measurements, a data acquisition switch unit (34970A, Keysight) is used to measure the capacitance between the MD-emitter-capacitor electrode and each MD-receiver-capacitor electrode, sequentially (one second for each measurement). As can be seen in Supplementary Fig. 8, when switch elements 0A-1B are connected via the data acquisition switch unit, we can measure d_4. And, if switch elements 0A-2B are connected, d_3 can be measured. T_{sen} can be estimated by using a digital multimeter (2400 Sourcemeter, Keithley) to measure the electrical resistance of the temperature sensor (see Supplementary Note 8). T_{TC} is measured by a K-type thermocouple attached to the heat sink with a thermally conductive adhesive (384™, Loctite). The data are collected repeatedly in the order of d_1, d_2, d_3, and (d_4, T_{sen}, and T_{TC}) until the MD emitter and the MD receiver are in contact. All experimental data are collected using LabVIEW software (National Instruments Corp.).

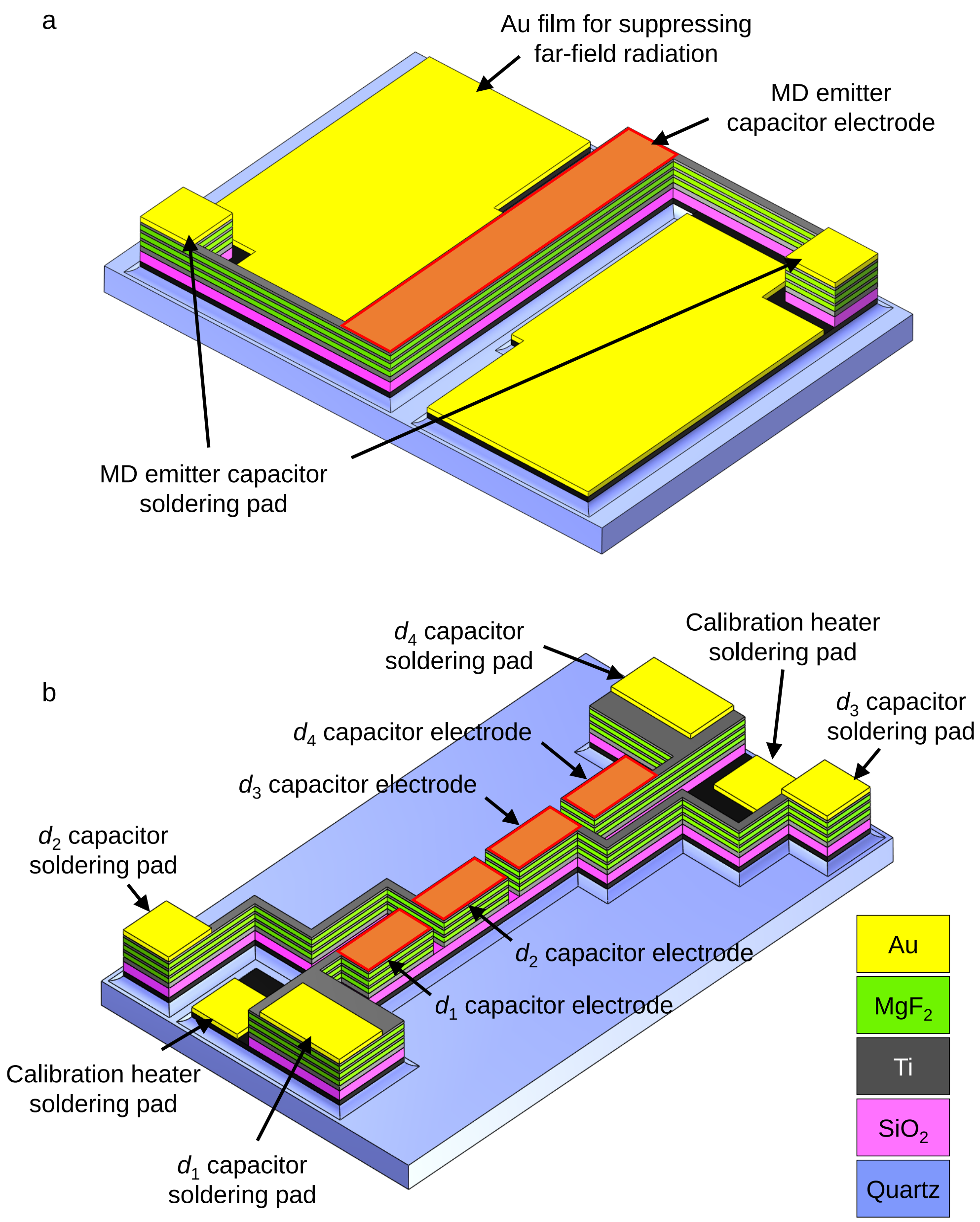

Supplementary Figure 7: Three-dimensional schematics of MEMS-fabricated microdevices. (**a**) Emitter part. MD emitter capacitor electrode, far-field radiation suppression Au film, and MD emitter capacitor soldering pads are described. (**b**) Receiver part. MD receiver capacitor electrodes ($d_{1\text{-}4}$ capacitor electrodes), MD receiver capacitor soldering pads ($d_{1\text{-}4}$ capacitor soldering pads), and calibration heater soldering pads are shown.

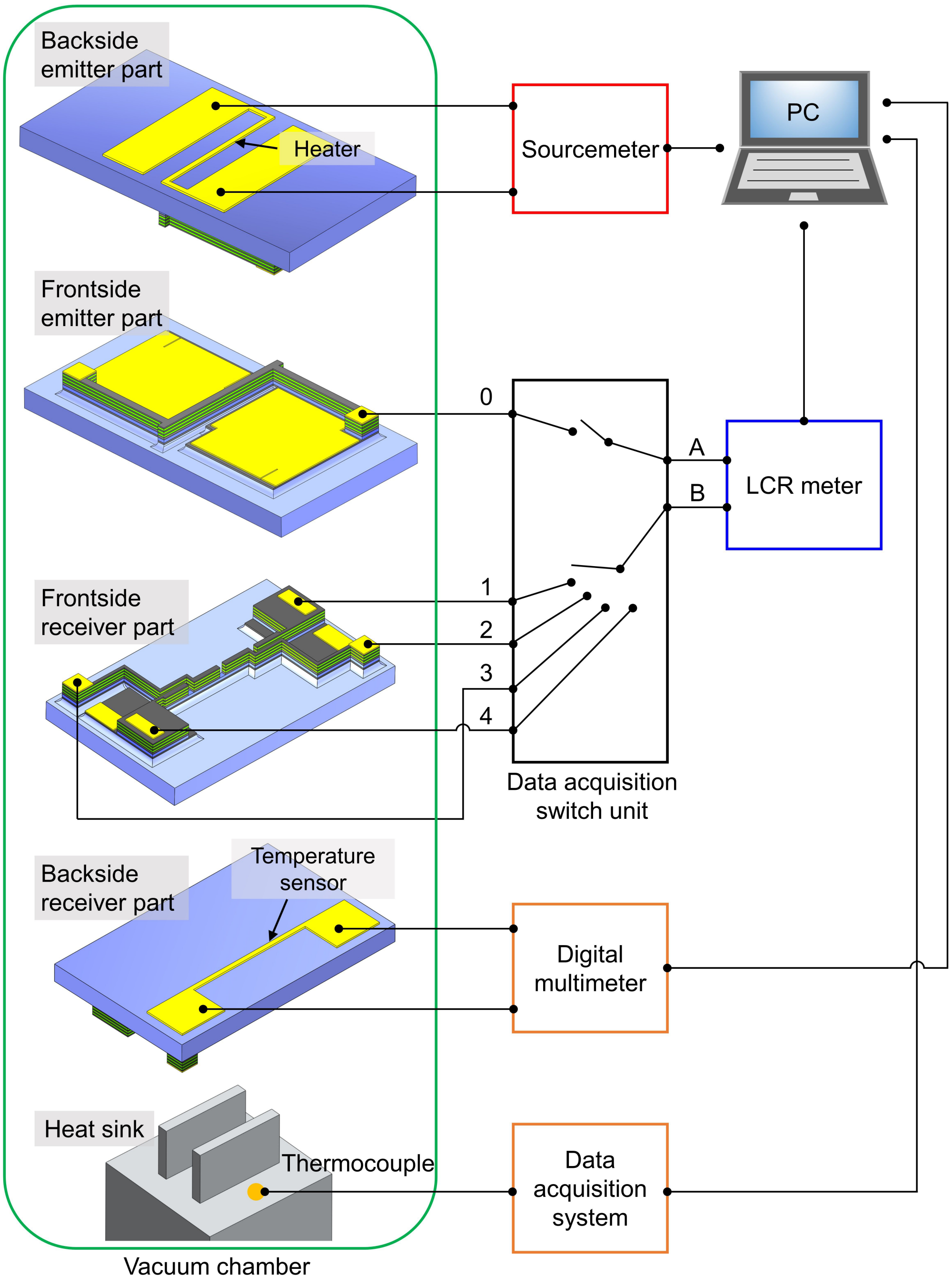

Supplementary Figure 8: Detailed experimental setup. Electrical resistance of heater and temperature sensor, capacitance between MD emitter capacitor electrode and MD receiver capacitor electrodes, and temperature of the heat sink are measured in this setup.

Supplementary Note 5. Capacitance calibration

In order to estimate the vacuum gap distance, the capacitance between the MD emitter and the MD receiver is measured. The topmost Ti layers of the MD emitter and the MD receiver (i.e., capacitor electrodes) are connected to an LCR meter (E4980AL, Keysight) via a vacuum feedthrough, a data acquisition switch unit (34970A, Keysight), and electric wires. The internal capacitances of the vacuum feedthrough, data acquisition switch unit, and electric wires lead to an increase of the measured capacitance. Accordingly, before estimating the vacuum gap distance between the MD emitter and the MD receiver from the measured capacitance, capacitance calibration should be conducted to obtain the following relation: $C_{\text{measured}}=C_{\text{actual}}+C_{\text{parallel}}$[1,2], where C_{measured} is the measured capacitance outside the vacuum chamber, C_{actual} is the actual capacitance between the MD emitter and the MD receiver, and C_{parallel} is the capacitance from the vacuum feedthrough, the switch unit, and the electric wires. To obtain C_{parallel}, we first increase the vacuum gap distance to larger than 1 mm, making C_{actual} a negligible value. At this stage, the corresponding capacitance, which can be considered as C_{parallel}, is measured. When the vacuum gap distance is 160 nm, C_{actual} is about six times larger than C_{parallel} for each segment. Consequently, the vacuum gap distance between the MD emitter and the MD receiver can be estimated from C_{actual}, obtained by subtracting C_{parallel} from C_{measured}.

Supplementary Note 6. Average vacuum gap estimation through Derjaguin approximation

We estimated the vacuum gap distance using the capacitance between the topmost Ti layers of the MD multilayers. The capacitance calibration should be performed as described in the preceding section (see Supplementary Note 5) to obtain the accurate vacuum gap distance. As described in the manuscript and in Supplementary Note 4, the capacitor electrode in the MD receiver is divided into four segments so as to measure four vacuum gaps as well as the curvature (and parallelism). The local distance d_i can be estimated using the relation: $d_i = \varepsilon A_i/C_i$, where A_i is the area of each MD receiver segment, C_i is the calibrated capacitance of each segment, and ε is the vacuum permittivity.

In Fig. 2b of the manuscript, it can be seen that the MD-multilayer surfaces are bowed, with curvature of 0.0029 m^{-1}. Because we can measure only the average radiative heat flux, Q_{avg} between MD multilayers (see Supplementary Note 9), the corresponding average vacuum gap distance, d_{avg} should be obtained from the four measured vacuum gap distances (d_1, d_2, d_3, and d_4). According to the calculated near-field heat flux, Q with respect to the vacuum gap distance, d (see Supplementary Fig. 9), the increment of Q is predicted to be proportional to $d^{-1.3}$ in a vacuum gap distance range of 100 nm to 1 µm. If we express the radiative heat flux, Q as $Q\{d(x)\} = ad(x)^{-1.3} + b$, we can use the Derjaguin approximation to obtain the average vacuum gap distance[3,4,5].

$$Q_{\mathrm{avg}} \times (WL) = \int_A Q\{d(x)\}dA = \int_0^L (ad(x)^{-1.3} + b)Wdx = aW \int_0^L d(x)^{-1.3}dx + bWL \tag{1}$$

where W is the width of MD receiver and L is the length of the MD receiver. Accordingly, we estimate the average vacuum gap distance, d_{avg} as

$$d_{\mathrm{avg}} = \left(\frac{1}{L}\int_0^L d(x)^{-1.3}dx\right)^{-1/1.3} \approx \left(\frac{1}{4}\sum_{i=1}^{4} d_i^{-1.3}\right)^{-1/1.3} \tag{2}$$

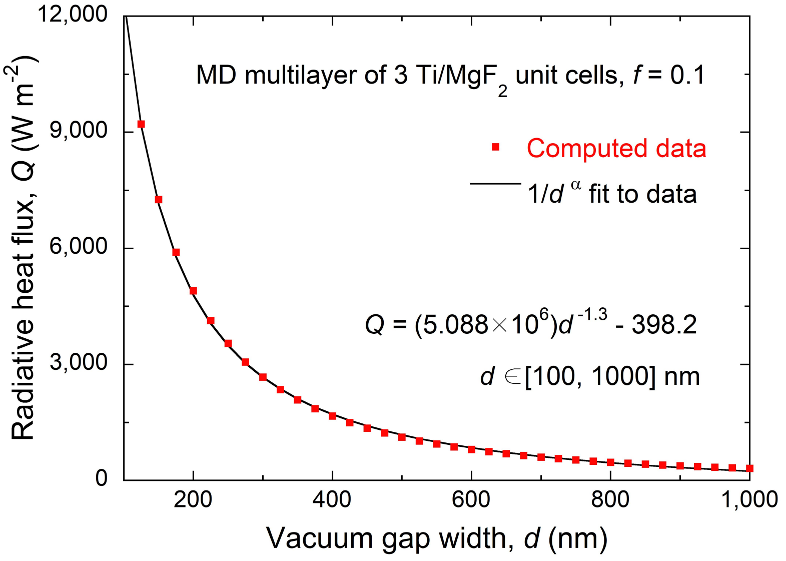

Supplementary Figure 9: Radiative heat flux analysis with respect to vacuum gap width (100 nm - 1 μm).

Supplementary Note 7. Confirmation of contact between MD emitter and MD receiver

It is crucial to monitor the contact between the MD emitter and the MD receiver because when these are in physical contact, the near-field radiative heat transfer can be overestimated due to unintended conduction heat transfer. The LCR meter (E4980AL, Keysight) can measure the capacitance between the MD-emitter-capacitor electrode and each of the MD-receiver-capacitor electrodes, allowing monitoring of the vacuum gap distance as well as providing a dissipation factor to determine whether the MD emitter and the MD receiver are in contact or not. The dissipation factor is an electrical property that measures the loss-rate of energy in a dissipative system[6,7]. In order to verify whether the dissipation factor can function as an indicator of contact between the MD emitter and the MD receiver, the electrical resistance, the capacitance, and the dissipation factor are measured in series while reducing the vacuum gap distance. If there is a protrusion on the MD-multilayer surface, the surface will be covered with a Ti layer, which is the last deposited layer. Therefore, when the MD emitter and the MD receiver are in physical contact (either surface to surface or surface to protrusion), the metal layers are electrically connected to each other and the corresponding electrical resistance between them has a finite value. When the MD emitter and the MD receiver are not in contact (i.e., open circuit), the electrical resistance is unable to be measured.

In Supplementary Fig. 10, as the capacitance increases (i.e., the vacuum gap distance decreases as the emitter moves close to the receiver), the dissipation factor remains close to zero. In this state, because the electrical resistance is unable to be measured, it can be considered that the MD emitter and the MD receiver are completely detached. Then, at the point when an increase of the dissipation factor is detected, the electrical resistance starts to have a finite value, which means that the MD emitter and the MD receiver are in contact. During the contact state, the measured negative capacitance is omitted, as shown in the Supplementary Fig. 10. By comparing the upper panel with the lower panel, it can be seen that every time the dissipation factor has a value larger than 1.0, the electrical resistance simultaneously has a finite value. Accordingly, it has been confirmed that all contacts can be observed via signals of the dissipation factor. Even when the MD emitter and the MD receiver are in contact, a large capacitance, which can be falsely regarded as indicating a very small vacuum gap, is sometimes measured. However, because the dissipation factor can indicate that the surfaces are in contact, the small estimated vacuum gap distance at this moment can be regarded as an erroneous value and is omitted from our final results. As the capacitance decreases (i.e., the vacuum gap distance increases), the measured dissipation factor and electrical resistance return to signals of non-contact. Therefore, in addition to the negative capacitance value, a dissipation factor larger than 1.0 can be used as a sign of physical contact

between the MD emitter and the MD receiver. The measurement of near-field radiative heat flux is conducted only when there is no physical contact (i.e., dissipation factor close to zero and positive capacitance).

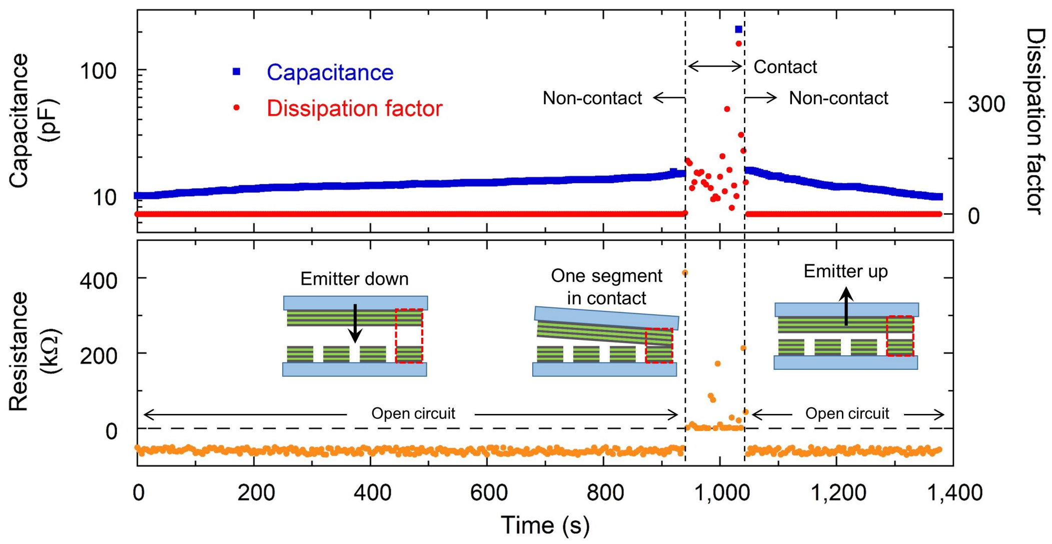

Supplementary Figure 10: Measurements of the capacitance, dissipation factor, and electrical resistance between MD emitter and MD receiver while changing vacuum gap distance.

Supplementary Note 8. Calibration of Au resistance temperature detectors

A resistance temperature detector (RTD) serving as a heater is deposited on the back of the emitter part (see Supplementary Fig. 2k) and an RTD serving as a temperature sensor is deposited on the back of the receiver part (see Supplementary Fig. 2k′). Utilizing a linear relationship of the electrical resistance and the corresponding temperature, the temperature can be estimated from the measured electrical resistance of the Au RTD. In order to obtain this linear relationship, calibration is conducted in a furnace using a K-type thermocouple. First, we put the Au RTD and the thermocouple in the furnace. Then, the temperature of the furnace is elevated to the desired temperature (135°C for heater calibration and 31°C for temperature sensor calibration). After that, while the furnace is slowly cooled to the room temperature, the electrical resistence of the Au RTD and the corresponding temperature are measured. The resistance of the Au RTD is measured using a digital multimeter (2400 SourceMeter, Keithley). Each Au RTD is calibrated within the temperature measurement range (25°C to 135°C for the heater and 25°C to 31°C for the temperature sensor). Supplementary Figure 11 shows the results of calibration of the two Au RTDs serving as the heater (Supplementary Fig. 11a) and the temperature sensor (Supplementary Fig. 11b). R-squared values are 1.000 for both.

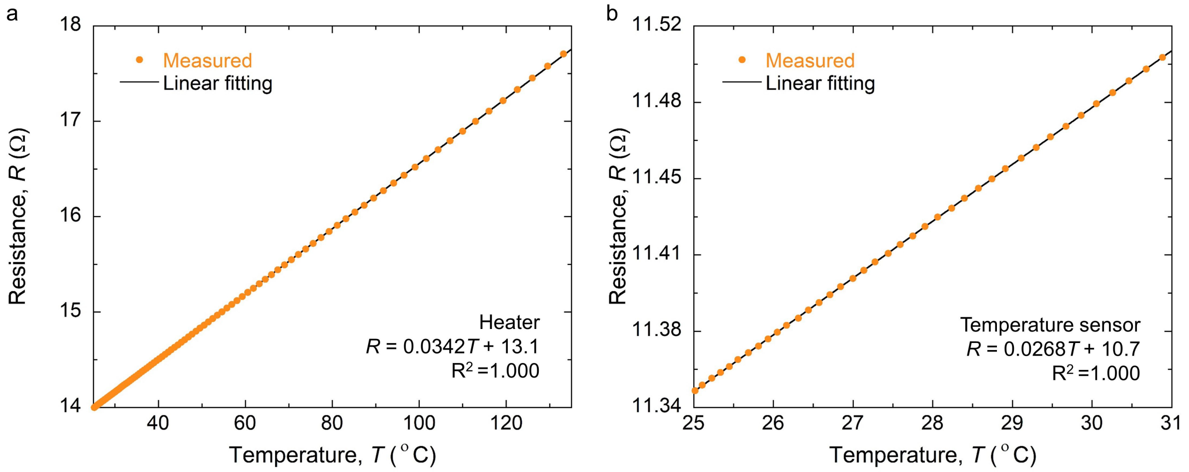

Supplementary Figure 11: Calibration results of Au resistance temperature detectors. (**a**) Heater (emitter part). (**b**) Temperature sensor (receiver part).

Supplementary Note 9. Calibration of heat flux

If the emitter part is at a position sufficiently far (about 4 μm) from the receiver part, then the radiative heat flux from the MD emitter to the MD receiver becomes minute. Contrarily, because the distance between the emitter and receiver parts, except for the MD emitter and the MD receiver, is already in the far-field regime due to the trenches on the substrates (trench height: 12 μm for the emitter part and 28 μm for the receiver part), the far-field radiation Q_{back1} can be treated as constant while reducing the vacuum gap between the MD emitter and the MD receiver; this can also be confirmed by calculation. During the experiment, we first placed the emitter part far away from the receiver part and then applied heating power to the heater. We controlled the heating power to keep the heater temperature constant. After confirming that the temperature of the heater is maintained at a constant set value, the emitter part is moved to the position (d ∼4 μm). At this position, various values of heat flux, Q are applied by Joule heating to the Ti calibration heater located under the SiO_2 insulation layer (see Fig. 3a of the manuscript). The corresponding temperature difference between the sensor and the thermocouple is recorded. The applied heat flux, Q and corresponding $T_{\mathrm{sen}} - T_{\mathrm{TC}}$ are plotted in Supplementary Fig. 12. Please note that the thermal resistance of the heat path between the Ti calibration heater and the top surface is negligible compared to that of the heat path between the Ti calibration heater and the temperature sensor, meaning that geometrical factor g is almost constant for heat-flux calibration condition and near-field experiment condition. Thus, the relationship between Q and $T_{\mathrm{sen}} - T_{\mathrm{TC}}$ can be safely used to estimate $Q_{\mathrm{e}\to\mathrm{r}}$ from the measured $T_{\mathrm{sen}} - T_{\mathrm{TC}}$ value. With our experimental setup, the absolute value of $Q_{\mathrm{e}\to\mathrm{r}}$ cannot be measured because we could not completely suppress the radiation from the MD emitter to the MD receiver during the calibration. Accordingly, only the increase of heat flux is measured while reducing the vacuum gap width. Considering the measurement uncertainty of the vacuum gap distance and the near-field radiative heat flux, we set a proper reference point of $d = 1000$ nm. It should be noted that $Q_{\mathrm{e}\to\mathrm{r}}$ at $d = 1000$ nm is much smaller than $Q_{\mathrm{e}\to\mathrm{r}}$ at $d = 160$ nm.

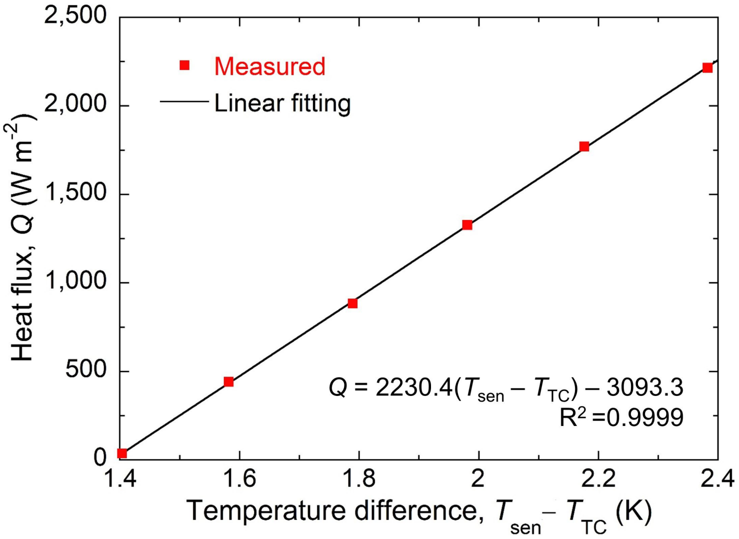

Supplementary Figure 12: Linear relationship between heat flux, Q, and the temperature difference between sensor and thermocouple, obtained from heat-flux calibration.

Supplementary Note 10. Near-field thermal radiation between Ti/dielectric multilayers

Supplementary Figure 13a depicts the near-field thermal radiation between Ti/dielectric multilayers, where dielectric material is MgF_2 (red dashed line) or lossless dielectric material ($\epsilon_d = 1.488$, blue dotted line). Although lossless dielectric material can neither emit nor absorb any radiation, similar radiative heat flux is obtained for both configurations. Also, the net radiative heat flux absorbed in each layer is plotted for vacuum gaps of 200 nm (Supplementary Fig. 13b) and 400 nm (Supplementary Fig. 13c). It is obvious that Ti layers are the main emitting layers and the enhancement in near-field thermal radiation is from the Ti layers, not from the MgF_2 layers. Although MgF_2 is a polar dielectric material that supports strong surface phonon polaritons, in this configuration (Ti/MgF_2 multilayers with Ti topmost layer), it rarely contributes to heat transfer enhancement because it is set as a supporting material (dielectric layer in metallo-dielectric multilayer), not as an emitting layer (metal-like layer in metallo-dielectric multilayer).

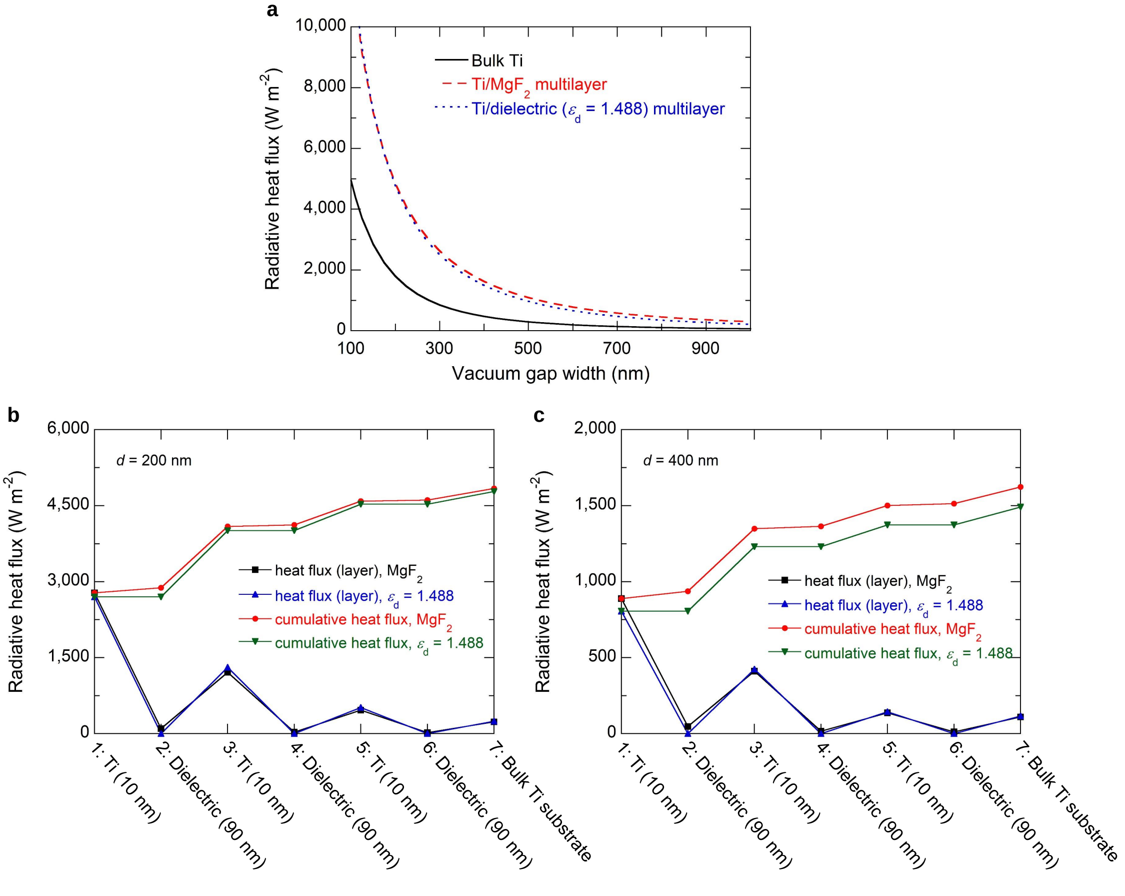

Supplementary Figure 13: (**a**) Near-field thermal radiative heat flux between bulk Ti media (black solid line), Ti/MgF_2 multilayers (red dashed line), and Ti/dielectric ($\epsilon_d = 1.488$) multilayers (blue dotted line). The net radiative heat flux absorbed in each layer of metallo-dielectric receiver and corresponding cumulative heat flux for vacuum gaps of (**b**) 200 nm and (**c**) 400 nm.

Supplementary Note 11. Uncertainty analysis

Measurement uncertainty of vacuum gap distance and near-field radiative heat flux is evaluated to estimate the precision of the experimental setup. Uncertainty is a parameter that represents the dispersion characteristics of measured values[8]. Uncertainty, u, is evaluated using the following equations.

$$u = \sqrt{\sum_{i=1}^{M}(c_i u_i)^2}, \qquad c_i = \frac{\partial f}{\partial x_i} \tag{3}$$

where u_i is the standard uncertainty and c_i is the sensitivity coefficient that describes how the output changes with respect to the variation of input variable, x_i. M is the number of input variables. In order to evaluate the standard uncertainty, uncertainty type A and type B are considered for every input variable. Type A, u_A, evaluates uncertainty by statistically analyzing a series of observations[8]; it can be calculated as follows.

$$u_\mathrm{A} = \sqrt{\frac{1}{N(N-1)}\sum_{k=1}^{N}(x_k - \overline{x})^2} \tag{4}$$

where N is the number of observations and $\overline{x}$ is the arithmetic mean of N observations. Type B, u_B, evaluates uncertainty in a manner other than statistical analysis of a series of observations[8]. Because we have confirmed that the measured data have a normal distribution, u_B can be evaluated by assuming that the measured data will be included in the 2 sigma interval (i.e., the probability that the observation is in the interval is 95.45%). Then, the standard uncertainty is defined by combining uncertainty type A and type B, as below[8].

$$u_i = \sqrt{u_{\mathrm{A},i}^2 + u_{\mathrm{B},i}^2} \tag{5}$$

In order to assess the measurement uncertainty of the near-field radiative heat flux, first, standard uncertainties for the electrical resistance of the temperature sensor ($<5 \times 10^{-4}$ Ω) and the temperature of the heat sink ($<7\times10^{-3}$ K) are calculated. The calibration results for the Au-RTD-temperature sensor (Supplementary Note 8) and the heat-flux calibration results (Supplementary Note 9) are used to determine the sensitivity coefficients. Accordingly, we found that the measurement uncertainty of the near-field radiative heat flux is <40 Wm^{-2}.

To obtain the measurement uncertainty of the vacuum gap distance, the standard uncertainty for the input capacitance ($<7\times10^{-14}$ F) was first calculated. The sensitivity coefficient is obtained from equation $d = \varepsilon A/C$,

where d is the vacuum gap distance, ε is a vacuum permittivity, A is the area of the vacuum gap distance sensor, and C is the measured capacitance. After calculating the four-vacuum-gap-distance (d_1, d_2, d_3, and d_4) measurement uncertainties, we evaluated the uncertainty of the average vacuum gap distance, d_{avg} between the MD emitter and the MD receiver. The vacuum gap distance measurement has an uncertainty of less than 5 nm.

The error bar of the measured near-field radiative heat flux value (y-axis error bar in Fig. 4 of the manuscript) and the error bar of the estimated vacuum gap distance (x-axis error bar in Fig. 4 of the manuscript) represents the combination of the measurement uncertainty and the standard deviation of multiple measurements. The x-axis error bars can hardly be seen because they are small compared to the size of the symbols.

Supplementary Note 12. Near-field thermal radiation between MgF_2/ dielectric multilayers

Supplementary Figure 14a depicts the net spectral heat flux between bulk MgF_2 media (black solid line) and that between MgF_2/dielectric multilayers (red dashed line). Here, dielectric material is assumed to be $\epsilon_d = 1.35$. For MgF_2/dielectric multilayer, three unit cells of MgF_2/dielectric layers are on a MgF_2 substrate where the thickness of both MgF_2 layer and dielectric layer is set to 200 nm. At vacuum gap of 200 nm, 21% enhancement over near-field thermal radiation between bulk MgF_2 media is achieved with MgF_2/dielectric multilayers. Also, it can be clearly seen in Supplementary Fig. 14b,c that the enhancement results from the coupling of surface phonon polaritons supported at the multiple interfaces (MgF_2/dielectric interface) within the MgF_2/dielectric multilayers. Please note that this is the same enhancement mechanism we have shown experimentally with Ti/MgF_2 multilayers.

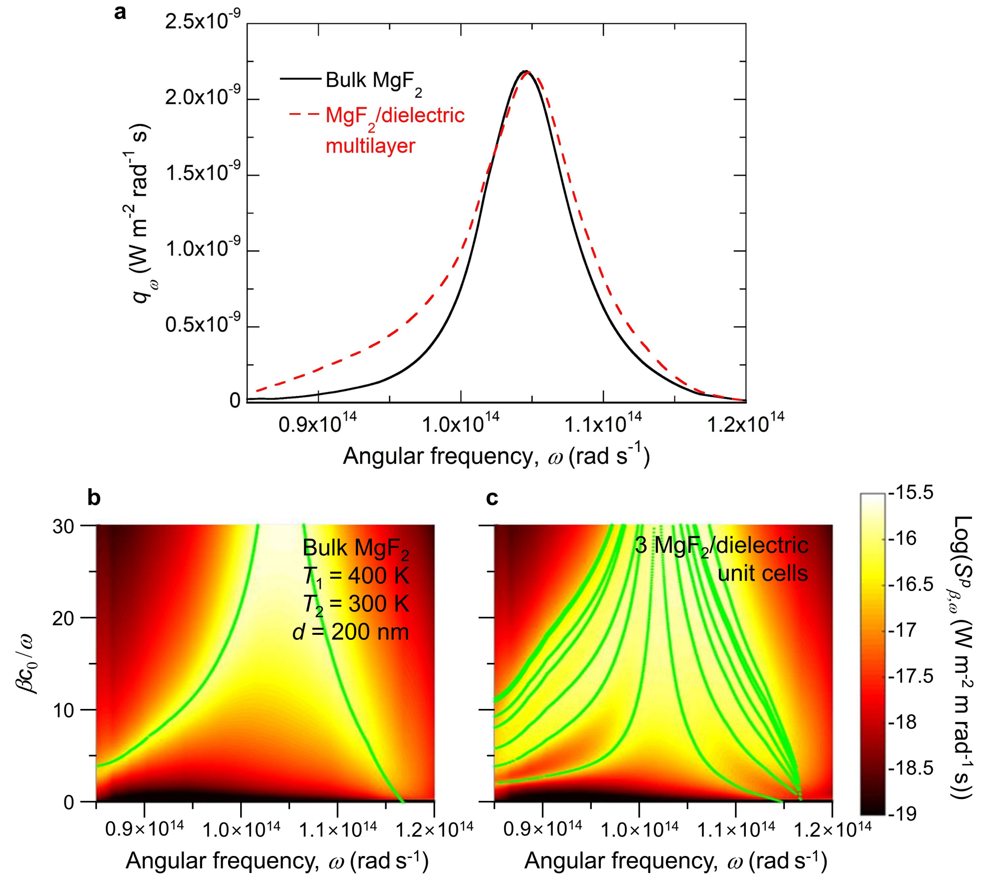

Supplementary Figure 14: (**a**) The net spectral heat flux between bulk MgF_2 media and between MgF_2/dielectric ($\epsilon_d = 1.35$) multilayers (three unit cells of MgF_2(200-nm-thick)/dielectric(200-nm-thick) on a MgF_2 substrate). The emitter and receiver temperatures are 400 K and 300 K, respectively. The vacuum gap width is 200 nm. (**b**) Computed $S^p_{\beta,\omega}$ between bulk MgF_2 media for vacuum gap distance of 200 nm and temperature of emitter and receiver conditions described in **a**. (**c**) As in **b**, but between MgF_2/dielectric multilayers.

Supplementary Note 13. Near-field thermal radiation

In order to achieve electrical insulation among MD receiver segments, a thick SiO_2 insulation layer is placed on the Ti calibration heater (see Supplementary Fig. 7b). In other words, on the quartz substrate, the Ti calibration heater is deposited first, then the SiO_2 insulation layer is deposited, and then the Ti/MgF_2 multilayer is deposited. To suppress the effect of the quartz substrate, the Ti calibration heater, and the SiO_2 insulation layer on the near-field thermal radiation between the MD emitter and the MD receiver, an additional 180-nm-thick Ti layer is placed between the SiO_2 layer and the Ti/MgF_2 multilayer. Supplementary Figure 15 shows the absorption distribution in each layer of the MD receiver at a vacuum gap of 1 μm. As can be clearly seen in Supplementary Fig. 15, the absorption within the quartz substrate, the Ti calibration heater, and the SiO_2 insulation layer is negligible (∼0.2% of the total absorption) due to the thick Ti layer beneath the MD structure. Because the penetration depth drops with decreasing vacuum gap distance[9], the contribution below the 180-nm-thick Ti layer to the radiative heat flux will be smaller as the vacuum gap distance decreases. Thus, when dealing with near-field thermal radiation, the fabricated structure can be safely assumed to be MD multilayer deposited on the bulk Ti substrate.

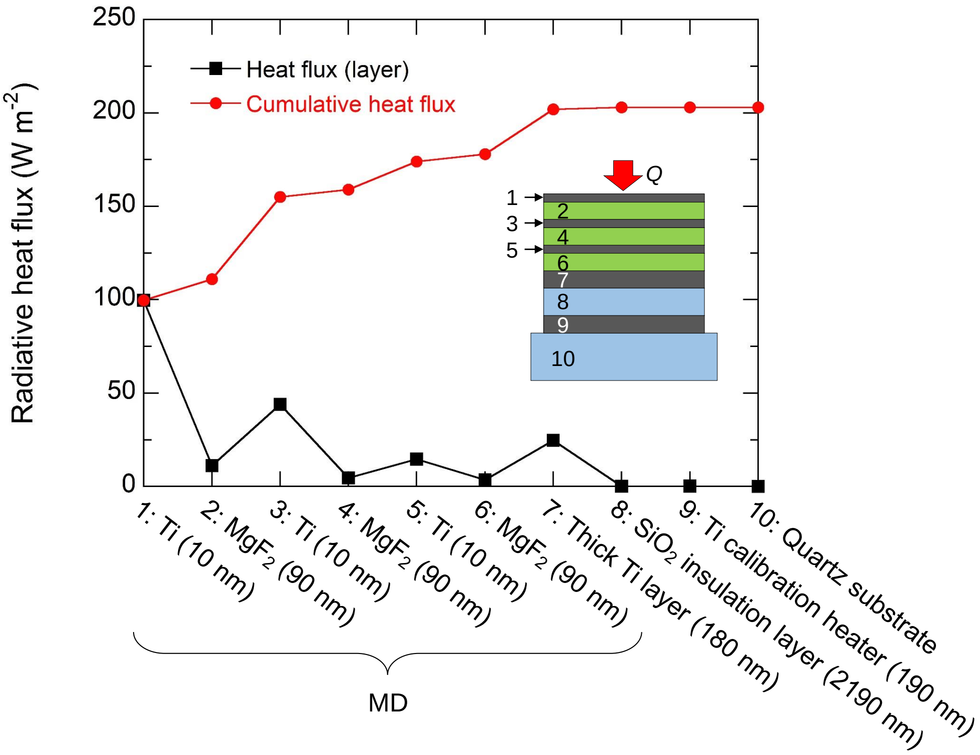

Supplementary Figure 15: Net radiative heat flux absorbed in each layer of MD receiver. Filling ratio, f: 0.1. Emitter temperature: 370 K. Vacuum gap distance: 1 μm.

Supplementary References

1. Lim, M., Lee, S. S. & Lee, B. J. Near-field thermal radiation between doped silicon plates at nanoscale gap. *Phys. Rev. B* **91**, 195136 (2015).

2. Ottens, R. S. *et al.* Near-field radiative heat transfer between macroscopic planar surfaces. *Phys. Rev. Lett.* **107**, 014301 (2011).

3. Song, B. *et al.* Radiative heat conductances between dielectric and metallic parallel plates with nanoscale gaps. *Nat. Nanotech.* **11**, 509-514 (2016).

4. Derjaguin, B. V., Abrikosova, I. I. & Lifshitz, E. M. Direct measurement of molecular attraction between solids separated by a narrow gap. *Quart. Rev. Chem. Soc.* **10**, 295-329 (1956).

5. Rousseau, E. *et al.* Radiative heat transfer at the nanoscale. *Nat. Photon.* **3**, 514-517 (2009).

6. Nilsson, J. W. *Electric Circuits* 2nd edn (Addison-Wesley, 1986).

7. Keysight E4980A/AL Precision LCR meter User's Guide,
Available at http://literature.cdn.keysight.com/litweb/pdf/E4980-90210.pdf

8. BIPM, IEC, IFCC, ILAC, ISO, IUPAC, IUPAP, and OIML. Evaluation of measurement data-Guide to the expression of uncertainty in measurement. JCGM 100: 2008 (2008).

9. Song, B. *et al.* Enhancement of near-field radiative heat transfer using polar dielectric thin films. *Nat. Nanotech.* **10**, 253-258 (2015).
